# Influence of ontogenetic development, temperature, and *p*CO_2_ on otolith calcium carbonate polymorph composition in sturgeons

**DOI:** 10.1038/s41598-021-93197-6

**Published:** 2021-07-06

**Authors:** Alison R. Loeppky, Luke D. Belding, Alex R. Quijada-Rodriguez, John D. Morgan, Brenda M. Pracheil, Bryan C. Chakoumakos, W. Gary Anderson

**Affiliations:** 1 Department of Biological Sciences, University of Manitoba, Winnipeg, MB Canada; 2 Department of Resource Management and Protection, Vancouver Island University, Nanaimo, BC Canada; 3 Environmental Sciences Division, Oak Ridge National Laboratory, Oak Ridge, TN Canada; 4 Neutron Scattering Division, Oak Ridge National Laboratory, Oak Ridge, TN Canada

**Keywords:** Climate-change impacts, X-ray crystallography, Developmental biology, Ichthyology

## Abstract

Changes to calcium carbonate (CaCO_3_) biomineralization in aquatic organisms is among the many predicted effects of climate change. Because otolith (hearing/orientation structures in fish) CaCO_3_ precipitation and polymorph composition are controlled by genetic and environmental factors, climate change may be predicted to affect the phenotypic plasticity of otoliths. We examined precipitation of otolith polymorphs (aragonite, vaterite, calcite) during early life history in two species of sturgeon, Lake Sturgeon, (*Acipenser fulvescens*) and White Sturgeon (*A. transmontanus*), using quantitative X-ray microdiffraction. Both species showed similar fluctuations in otolith polymorphs with a significant shift in the proportions of vaterite and aragonite in sagittal otoliths coinciding with the transition to fully exogenous feeding. We also examined the effect of the environment on otolith morphology and polymorph composition during early life history in Lake Sturgeon larvae reared in varying temperature (16/22 °C) and *p*CO_2_ (1000/2500 µatm) environments for 5 months. Fish raised in elevated temperature had significantly increased otolith size and precipitation of large single calcite crystals. Interestingly, *p*CO_2_ had no statistically significant effect on size or polymorph composition of otoliths despite blood pH exhibiting a mild alkalosis, which is contrary to what has been observed in several studies on marine fishes. These results suggest climate change may influence otolith polymorph composition during early life history in Lake Sturgeon.

## Introduction

Organisms that produce biogenic minerals through the process of biomineralization are widespread throughout the plant and animal kingdoms^1^. While the specific mechanisms and products of biomineralization are diverse among taxa, common approaches for regulating mineral production can be observed across phyla^2^. The function of biomineralized materials among organisms is also extensive. For example, some mollusks secrete hard calciferous shells that protect the organism, while mineralized bone materials provide the structural framework of the vertebrate skeleton^1^. Additionally, mobile organisms use calcified structures in their vestibular system, such as otoliths in fish—calcium carbonate (CaCO_3_) structures located in the inner ears—to detect sound, perceive gravity and sense linear and angular acceleration^3,4^. These calcifying organisms face particular challenges with climate change, especially in aquatic environments, where acidification results in a decline in the availability of carbonates (CO_3_^2−^), thus impacting the ability of the individual to precipitate CaCO_3_ structures^5^. Consequences on the processes of biomineralization in response to climate change depend on several factors specific to the organism, including biochemical pathways, physiological mechanisms, and the ecology of the organism^6^.

In fish, relatively little is known about the actual biogenesis and crystal growth mechanisms of otoliths resulting in a disparate gap in the fundamental understanding of otolith biomineralization compared to the number of studies that have examined their practical use for estimating age, growth, and habitat use^3,7^. Otoliths can be made of any of the three most common crystalline polymorphs of CaCO_3_ (i.e., aragonite, vaterite, calcite), each of which have varying unit cell densities and lattice structures^8–10^. Aragonite has the highest crystal growth rate^11^, which is conducive for creating heavy, dense, and constantly growing otoliths^12^. As such, the otoliths of most fishes, particularly teleosts, are primarily composed of aragonite^12,13^. Conversely, the otoliths of more primitive fishes, including acipenserids, have been classified for decades as being comprised entirely of the least dense, and structurally rarer polymorph vaterite^8,12^. Recent studies, however, demonstrated that otoliths of adult Lake Sturgeon, *Acipenser fulvescens*, contained significant proportions of calcite (~ 18–35%) in addition to vaterite^14^. As well, otoliths of age-0 Lake Sturgeon have been shown to be primarily composed of aragonite (> 60%) along with vaterite^15^. These results indicate that species in the earliest branch of Actinopterygii have the capacity to precipitate any form of CaCO_3_ polymorph, a mechanism of otolith biomineralization in fishes that previously was thought to only have evolved after the separation of teleosts from chondrostean (i.e., sturgeons, paddlefish) and holostean (i.e., gars, bowfins) lineages^13,16^.

Otolith polymorph composition appears to be both phylogenetically implicit and controlled by phenotypic responses to biological or environmental changes^8,16^. The otolith matrix contains various organic compounds (e.g., proteins, amino acids, collagens, proteoglycans), which guide the crystal nucleation controlling the polymorphism of CaCO_3_ crystallites^17–19^. In particular, otolin-1, a collagenous protein that stabilizes the mineral and organic compounds on the surface of otoliths, provides the framework for subsequent calcification during otolith growth^20^. Indeed, the presence of different compounds in the matrix (e.g., citrate, malate, pyruvate), which are controlled by protein metabolism, were identified to reduce calcification rates favouring the formation of calcite^17^. Additionally, the induced downregulation of *starmaker*, a gene responsible for regulating crystal growth, resulted in a switch from aragonite to calcite precipitation in zebrafish, *Danio rerio*^21^, suggesting the molecular control over matrix forming proteins, which can change throughout ontogeny, is an important factor in determining otolith polymorph composition^18^.

The environment can also impact otolith biomineralization. The classical process of CaCO_3_ crystallization is driven by the change in free energy required to transition from a supersaturated solution to a crystal^2,22^. The amount of free energy required to crystallize each polymorph decreases from calcite to aragonite to vaterite and, thus, the thermodynamic stability of each polymorph also decreases, respectively^8,23^. As such, temperature and environmental pH have direct impacts on supersaturation states and can lead to changes in the rate of crystallization and the precipitation of varying polymorphs of CaCO_3_^22^. Furthermore, when fish are exposed to environments with elevated carbon dioxide (*p*CO_2_), resulting in a decrease in environmental pH, a series of coordinated physiological responses occur to maintain internal pH gradients^24^; potentially influencing the solution chemistry of the endolymph^25^ and subsequently otolith morphology and/or crystal polymorph composition (reviewed in Ref.^26^). Few studies, however, have examined the effects of freshwater acidification on otolith development in fishes, none of which have been conducted on sturgeons. Freshwater systems regularly experience fluctuating temperature and environmental *p*CO_2_ due to the variation in sources of carbon from geologic dissolution, anthropogenic runoffs, decaying of organic matter, and seasonal variability^27^. As global temperatures and atmospheric *p*CO_2_ levels continue to rise, fluctuations in temperature and pH in freshwater environments are predicted to increase in both scale and frequency likely having a significant impact on the organisms that occupy these habitats^27,28^. As such, it is important to understand how elevated temperature and *p*CO_2_ might impact the morphology and development of freshwater fish otoliths, particularly at the vulnerable early life history stages when fish are typically more sensitive to environmental change^29,30^.

Studies examining otolith polymorph composition have to date focused primarily on the impacts of ocean acidification in marine fishes (listed in Ref.^26^), while to our knowledge, the effects of pH, temperature and/or ontogenetic development have not been empirically tested in any freshwater fish. In this study, we sought to provide novel insight on otolith phenotypic responses to both intrinsic (i.e., ontogeny) and extrinsic (i.e., environment) variables and how they relate to species-specific responses of otolith morphology in sturgeons. Specifically, we tested the hypotheses that (1) otolith polymorph composition varies throughout ontogeny and (2) water temperature and pH influence size and polymorphism of otoliths independent of ontogeny. To do this, percent polymorph composition was examined using X-ray microdiffraction throughout larval development in two sturgeon species, both of which are of critical concern throughout their North American ranges: Lake Sturgeon (*A. fulvescens*), a potadromous species that occupies the Great Lakes, Hudson Bay, and Mississippi River basins; and White Sturgeon (*A. transmontanus*), an anadromous species occupying the Sacramento-San Joaquin, Columbia, and Fraser River drainages. To investigate the influence the environment has on otolith morphology, otoliths collected from age-0 Lake Sturgeon were analyzed after long-term exposure (5 months) to varying temperature and *p*CO_2_ conditions that were chosen to mimic the projected changes increasing global temperature and atmospheric CO_2_ may have on freshwater systems in the natural range of Lake Sturgeon. Blood pH was also measured at the time of sampling to provide insight into the physiological mechanisms that influence otolith precipitation.

## Materials and methods

All procedures conducted on laboratory-reared fish were approved by the Animal Care Committee at the University of Manitoba (Permit F15-007) and Vancouver Island University (Permit AUP# 2015-01-H) in accordance with guidelines established by the Canadian Council on Animal Care. Otolith samples were transported to Oak Ridge National Laboratory in Tennessee, USA for analysis under CITES permit #18CA0004FONHQ.

### Experiment 1: Ontogenetic effects

To investigate changes in otolith polymorph composition throughout ontogenetic development of sturgeons, lab-reared fish used in this experiment were the artificially fertilized progeny of both Lake Sturgeon and White Sturgeon. Lake Sturgeon gametes were collected from wild-spawning adult fish captured in the Winnipeg River, Manitoba, Canada and White Sturgeon gametes were collected from resident adult males and females at the International Centre for Sturgeon Studies at Vancouver Island University in Nanaimo, British Columbia, Canada. The broodstock for these fish originated from the Lower Fraser River in Vancouver, BC. Seeding techniques and rearing procedures (including feeding) are provided in the Supplementary material (Supplementary Information 1.1). Both species were sampled on the same schedule throughout larval ontogeny corresponding to their respective times of hatch whereby eight larvae were randomly selected every 2 days from 12 to 20 days post hatch (dph) and then every 7 days until 69 dph (n = 88 fish/species). Sample sizes were chosen in consideration of statistical power as well as limitations on time and resources required for sample preparation and allotted instrument time for data quantification and further discussed in the supplementary information (Supplementary Information 1.2). At each sampling point, larvae were randomly selected and immediately euthanized in an overdose of tricane methanosulphate (MS-222) buffered with equal volumes of sodium bicarbonate, then transferred to 20 mL glass scintillation vials filled with 95% ethanol for preservation until analysis. Following techniques developed in Loeppky et al.^15^, both left sagittal and lapilli otoliths were then dissected (Supplementary Information 1.2) and transported to Oak Ridge National Laboratory (ORNL) in Tennessee, USA for quantification of otolith polymorph composition via X-ray microdiffraction (µXRD; S1.3). Quantitative phase analysis to determine the percent polymorph composition by weight of each otolith was conducted in the GSAS II open-source software package^31^ using the Rietveld method^32^.

### Experiment 2: Environmental effects

To investigate the effects of environmental temperature and pH on sagittal otolith size and polymorph composition of age-0 Lake Sturgeon, fish were reared from for six months in varying temperature and *p*CO_2_ treatments (Supplementary Table S1). Fertilized Lake Sturgeon eggs, collected as described in Experiment 1, were assigned to one of the following four experimental treatments: Control (15 °C, 1000 μatm *p*CO_2_), elevated *p*CO_2_ (15 °C, 2500 μatm *p*CO_2_), elevated temperature (22 °C, 1000 μatm *p*CO_2_) or elevated temperature + *p*CO_2_ (22 °C, 2500 μatm *p*CO_2_). To ensure the survival and proper development of larvae, all eggs were initially incubated at 15 °C. At 10 dph, fish in the elevated temperature treatments were gradually acclimated to increasing temperatures at a rate of 0.5 °C per day until 22 °C was achieved. Detailed description of the maintenance of experimental parameters are described in the supplementary information (Supplementary Information 1.1). Hatched larvae were held in 170 L fibreglass flow-through aquaria and reared in the same assigned experimental treatments for 150 days at which point temperature in all four treatment tanks was dropped to 3 °C (0.5 °C/day) to simulate an overwintering period (experimental *p*CO_2_ levels were maintained). After 40 days in the overwintering conditions, 8 fish/treatment (n = 32 fish total) were randomly sampled and immediately euthanized in an overdose of MS-222 buffered with equal volumes of sodium bicarbonate (250 mg/L) in their respective treatment water to maintain experimental CO_2_ tensions. As in Experiment 1, sample sizes were chosen in consideration of statistical power, resources for sample preparation and instrument time required to complete data collection. To account for differences among experimental rearing treatments, total body length (mm), fish mass (g) and blood pH were measured (Supplementary Information 1.4). The left sagittal otolith was then removed, imaged for size comparison (Supplementary Information 1.5), and analyzed via µXRD as described in Experiment 1.

### Statistical analyses

All analyses were conducted using SAS statistical software in JMP Pro^®^ version 15.2.1. Percent composition of otolith polymorphs were logit transformed prior to conducting analyses. For all data sets, response variables were first examined separately for normality using the Shapiro–Wilk W test and for homogeneity of variance using the Levene’s Test. In Experiment 1, only vaterite and aragonite were present in otoliths thus polymorph proportion in sagittal and lapilli otoliths (response variables, % weight) were examined using general linear models (GLMs) with species and developmental stage (dph) as fixed independent variables along with their interactions. Individuals were used as nested random factors to control for variation among fish. Post-hoc Tukey HSD tests were then conducted to determine which factor levels were different. In Experiment 2, regression analyses examining otolith area (mm^2^) as well as fish length (mm) and body mass (g) were determined using linear mixed models. Additionally, normalized differences (z-score) in otolith area (mm^2^), fish length (mm), and mass (g) among experimental treatments were tested using GLMs with temperature (°C) and *p*CO_2_ (µatm) as fixed independent factors followed by post-hoc Tukey HSD tests to determine which factor levels were different. To examine differences in vaterite, aragonite, and calcite composition (response variables, % weight) as well as blood pH, GLMs were used with temperature and *p*CO_2_ as fixed independent factors as well as their interactions. Individuals were used as nested random factors to control for variation within experimental treatments.

## Results

### Experiment 1

**Figure 1:**
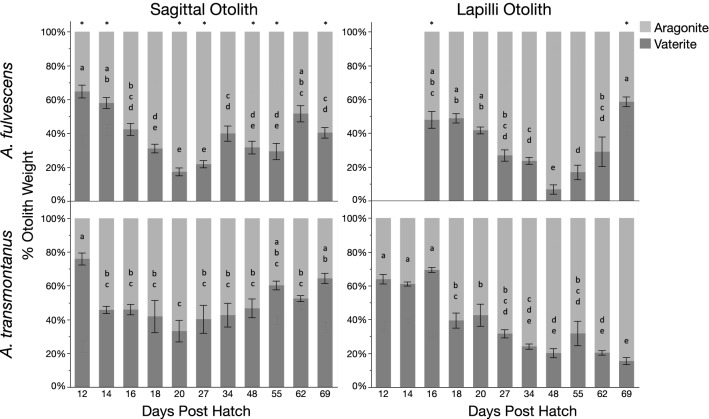
Mean (± s.e.m., n = 8 otoliths) percent polymorph composition (i.e., aragonite, vaterite) by weight of sagittal and lapilli otoliths collected throughout the ontogenetic development (i.e., days post hatch, dph) of Lake Sturgeon, *Acipenser* fulvescens, and White Sturgeon, *A. transmontanus*. Stars (*) indicate significant differences between species at developmental stage (i.e., days post hatch; dph) while letters indicate results of post-hoc Tukey HSD tests where developmental stages not sharing the same letter are significantly different. Lapilli otoliths of Lake Sturgeon are not included at 12 and 14 dph because they were too small to remove. Note the measured values are proportions thus the error bars apply to both aragonite and vaterite.

Environmental parameters (i.e., temperature, pH, alkalinity, hardness) during the rearing period for Lake and White sturgeon are presented in Supplementary Table S1. Both species had varying proportions of both vaterite and aragonite present in sagittal and lapilli otoliths that followed similarly changing patterns (Fig. 1). Statistical differences in polymorph composition among species and ontogenetic development are presented in Supplementary Table S2. Notably, aragonite composition was significantly higher in the sagittal otoliths of Lake Sturgeon compared to White Sturgeon while lapilli otoliths were not statistically different between species. Additionally, developmental stage (i.e., dph) had a significant effect on both sagittal and lapilli otolith polymorph composition with peak aragonite precipitation in sagittal otoliths occurring at 20 dph in both species (Fig. 1).

### Experiment 2

**Figure 2:**
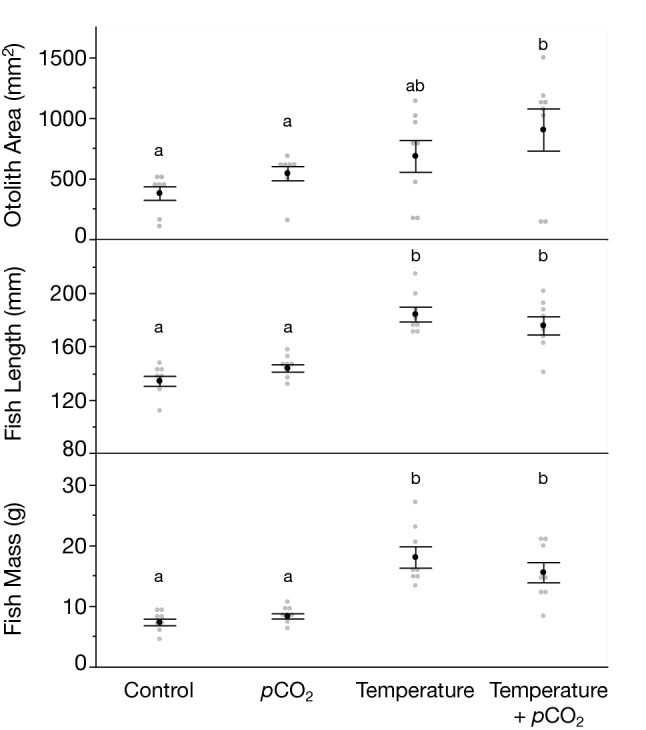
Mean (± s.e.m.) otolith area (mm^2^), fish length (mm), and fish mass (g) within each experimental treatment (i.e., control [15 °C, 1000 μatm *p*CO_2_], *p*CO_2_ [15 °C, 2500 μatm *p*CO_2_], temperature [22 °C, 1000 μatm *p*CO_2_], temperature + *p*CO_2_ [22 °C, 2500 μatm *p*CO_2_]). Grey points represent measurements for each individual (n = 8/treatment) and are jittered on the x-axis for visualization. Treatments not sharing the same letter are significantly different (ANOVA, p < 0.05).

**Figure 3:**
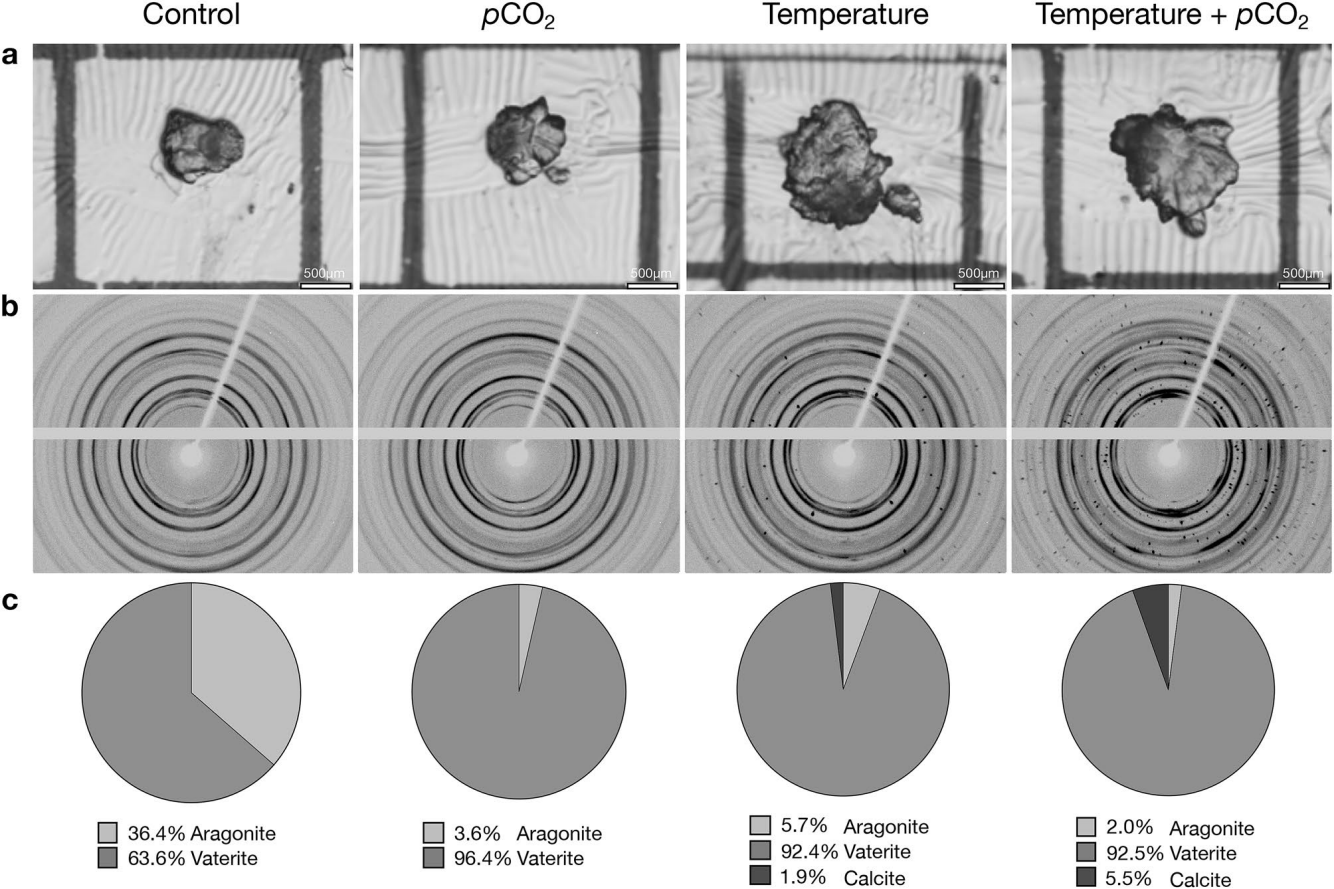
Examples of age-0 Lake Sturgeon, *Acipenser fulvescens*, otolith polymorph quantification results from single individuals within each experimental treatment (i.e., control [15 °C, 1000 μatm *p*CO_2_], *p*CO_2_ [15 °C, 2500 μatm *p*CO_2_], temperature [22 °C, 1000 μatm *p*CO_2_], temperature + *p*CO_2_ [22 °C, 2500 μatm *p*CO_2_]) including (**a**) images of sagittal otoliths at ×10 magnification, (**b**) Debye–Scherrer diffraction patterns collected using X-ray microdiffraction, and (**c**) percent polymorph composition (i.e., aragonite, vaterite, calcite) by otolith weight. Examples were chosen to highlight the variability in otolith polymorph composition observed among some individuals and the potential for large amounts of aragonite to be present in sturgeon otoliths that have previously long been considered to be comprised entirely of vaterite.

Environmental parameters (i.e., temperature, pH, alkalinity, *p*CO_2_) measured throughout the rearing and overwintering period are presented in Supplementary Table S1. Sagittal otolith area showed a positive relationship with both fish length and fish mass (Supplementary Fig. S1) with each variable being significantly influenced by experimental rearing temperature (Supplementary Table S2). In fact, all variables had the highest values in treatments that experienced elevated temperatures during the summer/fall rearing period (Fig. 2). This is most prominently observed in the size differences in otoliths presented in Fig. 3a. Interestingly, calcite precipitation was present only in otoliths collected from fish reared in elevated temperature treatments (i.e., temperature, temperature + *p*CO_2_; 4) with temperature being the only environmental variable that had a significant effect on polymorph precipitation (Supplementary Table S2). This is reflected in the diffraction patterns of otoliths from the elevated temperature and elevated temperature + *p*CO_2_ treatments where spots are clearly visible indicating the presence of large single calcite crystals (Fig. 3b,c). Despite not having an effect on otolith polymorph composition, environmental *p*CO_2_ significantly influenced blood pH with fish reared in elevated *p*CO_2_ treatments exhibiting a mild alkalosis (i.e., increase in blood pH; Supplementary Table S3).

## Discussion

While variation in otolith polymorph composition in response to ocean acidification is relatively well described in several marine teleosts, this is the first time the effects of ontogeny, temperature, and pH on the polymorphism of otoliths has been experimentally manipulated providing important primary information on the development otoliths in a freshwater acipenserid at the larval and juvenile life stages. Early growth and development of specific organs has long lasting effects on the phenotype of the structure in all organisms, not least fishes. Given otoliths are considered inert, once the matrix and crystal polymorphs are deposited, there is no reabsorption of the otolith material^3^. Therefore, the development of otoliths set during early ontogeny may have significant impacts on future hearing and orientation abilities, which are key senses that are required for habitat choice, predator avoidance, and foraging. Here we have described changes in polymorph composition in the otoliths of Lake Sturgeon and White Sturgeon during early ontogeny and demonstrate significant effects of the environment on otolith development in age-0 Lake Sturgeon. Given the novelty of this research on both the intrinsic and extrinsic variables influencing the precipitation of acipenserid otoliths, we present here our interpretations of the data and the hypotheses we propose, which will guide the direction of future studies.

### Ontogenetic effect

By examining the early ontogeny of otoliths in Lake and White Sturgeon, we followed the progression of otolith formation in two species with disparate life history characteristics. In the wild, spawning for each species occurs in freshwater habitats and, thus, the development of eggs and larvae occurs in broadly comparable freshwater environments. Both sagittae and lapilli otoliths collected from Lake and White Sturgeon showed similarly varying proportions of percent vaterite and aragonite throughout the developmental period with significant differences in overall mean percent polymorph composition reported between species in sagittal otoliths only (Fig. 1, Supplementary Table S2). During the rearing period, both species were held independently in constant conditions, however, the environmental parameters experienced by each species were different. Temperature was higher during larval rearing for Lake Sturgeon and although not explicitly measured in this study, City of Winnipeg and Nanaimo water quality reports (winnipeg.ca; nanaimo.ca) indicate differences in total metal content, alkalinity, and water hardness between sources (Supplementary Table S1). Water hardness and alkalinity in particular were considerably different whereby Lake Sturgeon were reared in moderately hard and alkaline water while White Sturgeon were reared in soft and lower alkalinity water. Both measurements reflect the amount of carbonates present in the system, which in turn affects the development of larval fishes, in particular their calcified structures^33^. Additionally, temperature impacts the solubility of ions and has been observed to increase metal uptake and deposition mechanisms in Lake Sturgeon^34^. Consequently, with the higher availability of Ca^2+^ and carbonate ions, changes in supersaturation in the endolymph of Lake Sturgeon may have been affected thus impacting the crystallization rates of the otoliths and increasing the precipitation of the denser polymorph aragonite, although further research is necessary to test this hypothesis in sturgeon.

The similarities observed in the patterns of percent vaterite and aragonite changes throughout ontogeny among both species, with relatively low variation (Fig. 1), suggest genetic control of CaCO_3_ polymorph crystallization in sturgeons. The organic matrix, which controls the nucleation, shape, and orientation of crystallites, forms when otolith soluble-matrix proteins are secreted into the endolymph by specialized cells in the saccular epithelium^35^. As such, changes in the metabolism of key amino acids affects the synthesis of proteins that regulate the rate of calcification and, thus, the polymorphism of CaCO_3_ crystals^20,35,36^. Interestingly, peak aragonite composition in sagittal otoliths occurred simultaneously at 20 dph in both species, coinciding with the complete transition to exogenous feeding, at which point vaterite precipitation increased. At hatch, sturgeon rely on yolk sac reserves that are high in amino acids as sources of metabolic fuel, which are essential for the growth and development of larvae^37^. As such, during yolk resorption, free amino acid availability, necessary for protein synthesis, steadily diminishes until first feeding^37^. This in turn would affect the rate of protein synthesis in the saccular membrane thus impacting the composition of the otolith organic matrix within the saccule^38^. A similar occurrence was observed in multiple marine species where diet correlated with changes in otolith shape attributed to variation in the quantity and composition of proteins in prey^39^.

The difference in protein content of the diet provided to both species at first feeding may also have been a factor in the overall mean differences in vaterite proportions observed in the sagittal otoliths throughout development. Lake Sturgeon larvae were initially fed a diet of artemia nauplii, which contain ~ 60% crude protein (reedmariculture.com), while White Sturgeon were fed EWOS commercial aquaculture feed containing ~ 54% crude protein (cargill.com). Although not directly measured in our study, the difference in protein content, along with other essential nutrients (e.g., lipids, carbohydrates and minerals), likely affected the proportion of water-soluble (associated with mineral phase) and -insoluble (associated with structural foundation for crystal growth) proteins that were synthesized in the endolymph^40^. One such protein is otolin, a chemically unique protein specific to otoliths that is characterized by a high abundance of acidic amino acids^34,36,38^. These acidic amino acids contribute to otolith mineral formation by concentrating Ca^2+^ ions and CO_3_^2-^ molecules thus inducing supersaturation of the system, which is required for crystallization to occur^2,40^. As such, the amount of otolin present influences the probability of crystal nucleation and consequently the growth of otoliths^2,36^. The lapilli otoliths of both species followed similar trends of increasing aragonite composition until 48 dph. After 48 dph, increased precipitation of aragonite continued in White Sturgeon, while a switch to vaterite precipitation is clearly demonstrated in Lake Sturgeon lapilli otoliths. Interestingly, it was during this transition period 50 dph that Lake Sturgeon larvae were introduced to bloodworm, which contain a minimum crude protein of only 6% (hikari.com). This transition was gradual whereby the proportion of bloodworm was increased by 10% every two days and as such the residual effects on otolith formation were likely delayed. Overall these results suggest that diet may have a significant influence on matrix proteins that control CaCO_3_ precipitation in sturgeons, however, further investigation of protein expression in the saccule is needed to confirm this hypothesis.

Despite such large proportions of aragonite being present in all otoliths at the larval stages of sturgeon development in this study, as well as in Loeppky et al.^15^, no aragonite was reported in the adult Lake Sturgeon otoliths measured by Pracheil et al.^14^ when examined via neutron diffraction. As sturgeon age and grow, and consequently their otoliths grow, vaterite and calcite appear to be the primary CaCO_3_ polymorphs being precipitated. As such, the proportion of aragonite that is crystallized during the larval stages may eventually fall below the detectable limits of Rietveld refinement in the larger mass otoliths of adults.

### Environmental effects

The effects of the environment on otolith morphology have thus far focused primarily on teleosts (e.g., Refs.^24,27,39^). In this study, we examined for the first time the combined effects of temperature and pH on otolith area and polymorph composition. Indeed, otolith area was significantly influenced by rearing treatment and had a positive relationship with both fish length and mass (Fig. 2, Supplementary Fig. S2). This is similar to teleosts where, in general, otolith growth is proportional to somatic growth and is related to the metabolism of the individual^41,42^. Perhaps not surprisingly, temperature was the driving factor in the size differences among experimental rearing environments with otolith area, fish length, and mass being highest in the elevated temperature treatments. This is particularly evident in the size differences in otoliths sampled among treatments (Fig. 3a).

**Figure 4:**
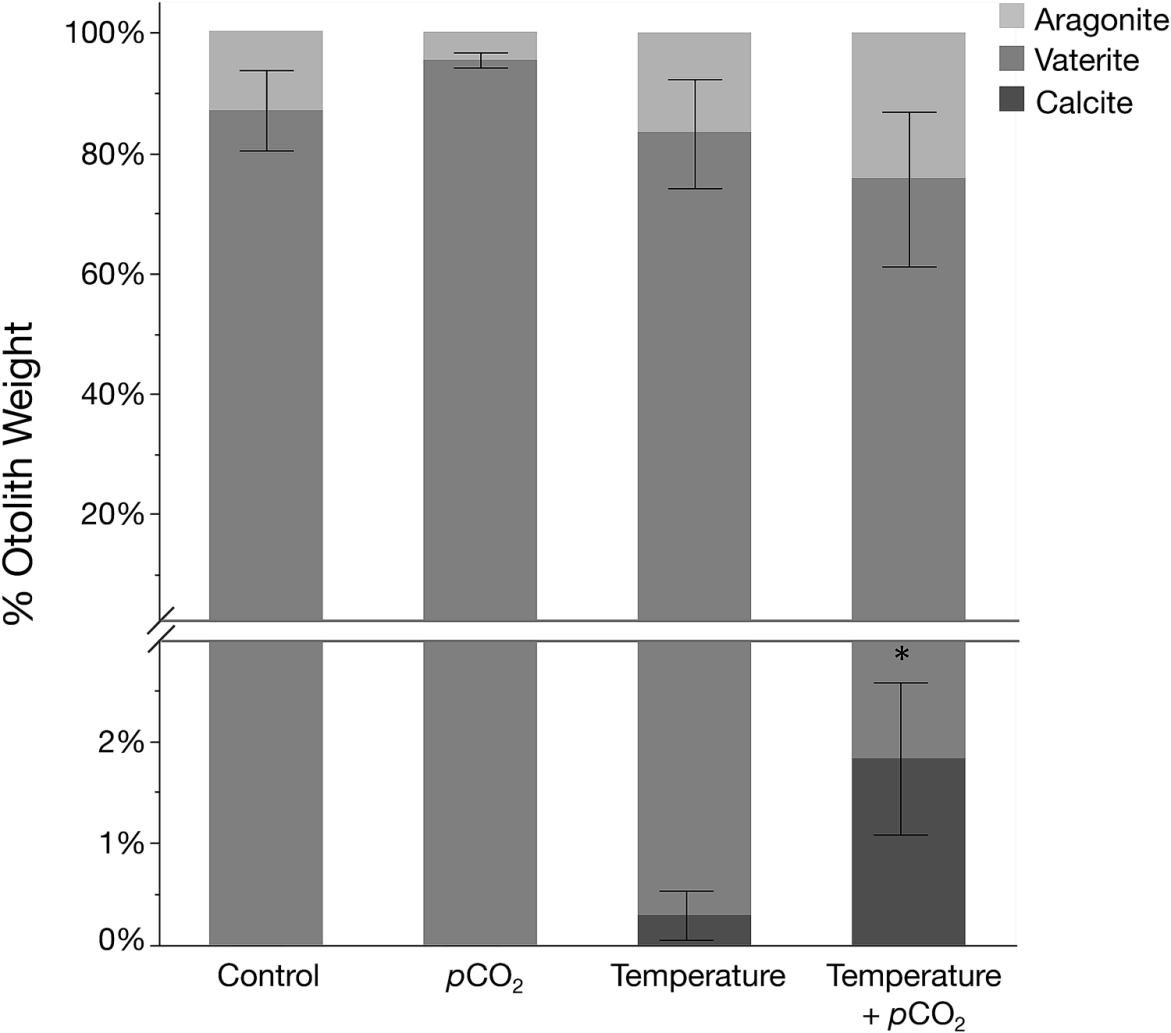
Mean (± s.e.m., n = 8 otoliths) percent polymorph composition (i.e., aragonite, vaterite, calcite) by weight of sagittal otoliths collected from age-0 Lake Sturgeon, *Acipenser fulvescens*, within each experimental treatment (i.e., control [15 °C, 1000 μatm *p*CO_2_], *p*CO_2_ [15 °C, 2500 μatm *p*CO_2_], temperature [22 °C, 1000 μatm *p*CO_2_], temperature + *p*CO_2_ [22 °C, 2500 μatm *p*CO_2_]). Note the break in y-axis corresponds to a change in scale. *Indicates calcite composition is significantly different among experimental treatments where calcite was present (ANOVA, < 0.05).

Interestingly, calcite precipitation was only observed in the otoliths also sampled from fish reared in the elevated temperature treatments (Fig. 4). This is reflected in the X-ray diffraction patterns of these otoliths, which displayed spots (i.e., single-crystal Bragg peaks) whereas finer grained vaterite and aragonite produced Debye–Scherrer rings (Fig. 3b). The spots are indicative of relatively large single-crystal calcite grains (> 100 µm up to mm size) implying a lower nucleation rate as compared to aragonite and vaterite, suggesting temperature had an effect on the crystallization process in the otoliths of the fish in our study. Vaterite is a precursor to denser aragonite and calcite CaCO_3_ polymorphs, the pathways of which are controlled by both thermodynamic and kinetic reactions^43^. Therefore, increasing temperature, increases the rate of these reactions by raising the kinetic energy available to the system. Temperature also influences the free energy response of minerals by affecting their chemical potential and solubility making Ca^2+^ and CO_3_^2−^ more readily available to be transported by enzymes^44^. The rate of transport into the saccular epithelium is also increased by temperature, which in turn would also increase CaCO_3_ accretion and the growth rate of otoliths^44,45^. Additionally, the precipitation of large single calcite crystals may have been controlled by differential expression of otolith matrix proteins in the saccular membrane. Given the strong connection of biomineralized structures with organic matrices that act as templates for crystallization, the organic matrix in otoliths is presumably the main controller of the orientation and size of crystallites being formed^18,36^. Temperature also directly influences protein synthesis and, thus, impacts the composition of proteins in the saccule. Similarly, the proportion of water-soluble and -insoluble proteins were disparately affected by temperature increases in the otoliths of juvenile cod, *Gadus morhua*, impacting the density and, thus, microstructure of their otoliths^46^. Regardless of the mechanism, results from our study suggest calcite precipitation may be temperature dependent in age-0 Lake Sturgeon.

Despite *p*CO_2_ reportedly having an influence on the otolith morphology of multiple marine teleosts (e.g., Refs.^29,30,47,48^), environmental *p*CO_2_ (and therefore pH) had no effect on otolith size or polymorph composition in our study. Acid–base regulation is a critical physiological process as inappropriate maintenance will influence the overall performance of the individual by impacting the functionality of proteins and other essential macromolecules^49^. Sturgeons are considered to be particularly tolerant to low pH environments and are able to tightly regulate intra- and extra-cellular acid–base balance^24,49^. These highly sophisticated physiological mechanisms likely evolved to historical extremely high *p*CO_2_ environments when global climates more closely resembled tropical aquatic systems (~ 250–300 million years ago; Refs.^24,50^). Interestingly, the blood pH measurements collected at the time of sampling indicated the Lake Sturgeon in our study exhibited a mild alkalosis when compensating for the elevated levels of *p*CO_2_ in the environment (Supplementary Table S3). This is evidenced by the significant increase in blood pH (i.e., more basic) in comparison to fish that were reared in control *p*CO_2_ environments. The mild alkalosis observed in our study may have been a factor of the chronic exposure to elevated *p*CO_2_ that the age-0 Lake Sturgeon experienced as typically an initial rapid acidosis in fishes (decrease in blood pH) is gradually corrected by metabolic alkalosis to return blood to a normal pH^24^. As such, it is likely that for the majority of time during development, the highly regulated distal and proximal endolymph pH, which controls the ionic state in the environment that the otoliths are in direct contact with, would have been buffered against external *p*CO_2_ conditions^19^. Although not statistically significant, the variability in otolith polymorph composition was reduced in the *p*CO_2_ treatment suggesting greater control over CaCO_3_ precipitation (Fig. 4). Had the fish in our study been exposed to greater differences in environmental *p*CO_2_ there likely would have been greater impacts on otolith morphology. While the interaction between the environment and the specific mechanisms of phenotypic expression of otolith polymorphs remains unclear, our results suggest environmentally relevant changes in temperature, over pH, plays a primary role in the phenotypic plasticity of otolith crystallization in Lake Sturgeon.

## Conclusions

Overall, this study provides a novel demonstration of the interplay between biological and environmental controls on otolith phenotypes. We showed that otolith polymorph composition varies similarly throughout early Lake and White sturgeon ontogeny, suggesting otolith polymorph composition is under genetic control and may also be correlated to diet at this life stage. On the other hand, temperature also influenced otolith phenotypic plasticity, increasing otolith size and inducing the precipitation of large single calcite crystals.

Our findings have several implications for fish conservation. Information on polymorph composition through early ontogeny is important for locating the initial seeding of sturgeon otoliths, which may help with identification of the core, or first formed, region in otoliths of adult fish. The post-hatch otolith core contains elemental signatures of the natal areas of fish and can be useful for identifying critical habitats in need of protection^51^. The consequences of variation in polymorph composition on the behavioural performance of sturgeons (i.e., hearing, orientation, foraging) remains unknown and is integral to understand the survivability of individuals with malformed otoliths. Fish reared in aquaculture settings are typically subjected to controlled environments where conditions are markedly different to those experienced by wild conspecifics. Despite this, the release of hatchery-reared fish is a commonly used conservation technique, often applied to increase sturgeon stocks. Fish hatcheries often encourage enhanced growth by feeding enriched diets, increasing water temperatures, and using continuous light to encourage rapid growth rates^52^. The increased growth rate in hatchery reared fish likely impacts otolith matrix proteins resulting in the abnormal crystallization of otoliths in farmed fish and has been shown to result in hearing impairment in farmed salmonids^52^. For conservation hatcheries, where the primary goal is to produce offspring that are capable of survival post hatchery release and contribute to future populations^53^, it is important to ensure rearing practices do not have adverse effects on the development of individuals. If changes in otolith size and composition (affecting density) have adverse effects on the hearing and navigation capabilities of sturgeons, this may impact the survivability of stocked individuals once introduced into wild environments. Further research, however, is required to understand the behavioural effects malformed otoliths have on fishes.

To understand the underlying processes of otolith morphology and CaCO_3_ polymorph determination, investigating the molecular mechanisms involved in protein expression in the saccule is essential. While these mechanisms are described to some degree in teleosts (e.g., Refs.^21,54–56^), the equivalent mechanisms in acipenserids are unknown. Sturgeon are primitive fishes often considered living fossils, suggesting that evolutionary change in this group is relatively slow. Given their phylogenetically more distant status, identifying the proteins involved in the synthesis of the organic matrix in chondrosteans, and the mechanisms involved in their expression, will offer new insights into the evolution of hearing and balance structures among fish species and potentially higher vertebrates.

## Supplementary Information

Supplementary Information.

## Data Availability

https://datadryad.org/stash/share/y80uRCPVOWBAmzTEVryA8zR5cUr0kUGzoYg7M-Yij8c.

## References

[1] Lowenstam, H. A. *On Biomineralization*. (ed. Weiner, S.) (Oxford University Press, 1989).

[2] De Yoreo JJ, Vekilov PG (2003). Principles of crystal nucleation and growth. Rev. Mineral. Geochem..

[3] Campana SE (1999). Chemistry and composition of fish otoliths: Pathways, mechanisms and applications. Mar. Ecol. Prog. Ser..

[4] Popper AN, Ramcharitar J, Campana SE (2005). Why otoliths? Insights from inner ear physiology and fisheries biology. Mar. Fresh. Res..

[5] Gattuso J-P, Buddemeier RW (2000). Calcification and CO_2_. Nature.

[6] Ries JB, Cohen AL, McCorkle DC (2009). Marine calcifiers exhibit mixed responses to CO_2_-induced ocean acidification. Geology.

[7] Campana SE (2005). Otolith science entering the 21st century. Mar. Freshw. Res..

[8] Gauldie RW (1993). Polymorphic crystalline-structure of fish otoliths. J. Morphol..

[9] Chakoumakos BC, Pracheil BM, Koenigs RP, Bruch RM, Feygenson M (2016). Empirically testing vaterite structural models using neutron diffraction and thermal analysis. Sci. Rep..

[10] Christy AG (2017). A review of the structures of vaterite: The impossible, the possible, and the likely. Cryst. Growth Des..

[11] Gauldie RW, Nelson DGA (1988). Aragonite twinning and neuroprotein secretion are the cause of daily growth rings in fish otoliths. Comp. Biochem. Phys. A.

[12] Schulz-Mirbach T, Ladich F, Plath M, Heß M (2019). Enigmatic ear stones: What we know about the functional role and evolution of fish otoliths. Biol. Rev..

[13] Carlstrom DD (1963). Crystallographic study of vertebrate otoliths. Biol. Bull..

[14] Pracheil BM (2017). Sturgeon and paddlefish (Acipenseridae) sagittal otoliths are composed of the calcium carbonate polymorphs vaterite and calcite. J. Fish Biol..

[15] Loeppky AR, Chakoumakos BC, Pracheil BM, Anderson WG (2019). Otoliths of sub-adult Lake Sturgeon *Acipenser fulvescens* contain aragonite and vaterite calcium carbonate polymorphs. J. Fish Biol..

[16] Pracheil BM, George R, Chakoumakos BC (2019). Significance of otolith calcium carbonate crystal structure diversity to microchemistry studies. Rev. Fish Biol. Fish..

[17] Kitano Y, Hood DW (1965). The influence of organic material on the polymorphic crystallization of calcium carbonate. Geochim. Cosmochim. Acta..

[18] Thomas ORB (2019). The inner ear proteome of fish. FEBS J..

[19] Payan P, De Pontual H, Boeuf G, Mayer-Gostan N (2004). Endolymph chemistry and otolith growth in fish. C. R. Palevol..

[20] Murayama E, Herbomel P, Kawakami A, Takeda H, Nagasawa H (2005). Otolith matrix proteins OMP-1 and Otolin-1 are necessary for normal otolith growth and their correct anchoring onto the sensory maculae. Mech. Dev..

[21] Sollner C (2003). Control of crystal size and lattice formation by starmaker in otolith biomineralization. Science.

[22] Ruiz-Agudo E, Putnis C, Rodriguez-Navarro C, Putnis A (2011). Effect of pH on calcite growth at constant aCa^2+^/aCO_3_^2^^−^ ratio and supersaturation. Geochim. Cosmochim. Acta..

[23] Dalas E, Kallitsis J, Koutsoukos PG (1988). The crystallization of calcium carbonate on polymeric substrates. J. Cryst. Growth.

[24] Brauner C, Baker D, Glass M, Wood S (2009). Patterns of acid–base regulation during exposure to hypercarbia in fishes. Cardio-Respiratory Control in Vertebrates Comparative and Evolutionary Aspects.

[25] Ishimatsu A, Hayashi M, Kikkawa T (2008). Fishes in high-CO_2_, acidified oceans. Mar. Ecol. Prog. Ser..

[26] Holmberg RJ (2019). Ocean acidification alters morphology of all otolith types in Clark's anemonefish (*Amphiprion clarkii*). PeerJ.

[27] Hasler CT (2018). Biological consequences of weak acidification caused by elevated carbon dioxide in freshwater ecosystems. Hydrobiologia.

[28] Ficke AD, Myrick CA, Hansen LJ (2007). Potential impacts of global climate change on freshwater fisheries. Rev. Fish Biol. Fish..

[29] Munday PL, Gagliano M, Donelson JM, Dixson DL, Thorrold SR (2011). Ocean acidification does not affect the early life history development of a tropical marine fish. Mar. Ecol. Prog. Ser..

[30] Bignami S, Enochs IC, Manzello DP, Sponaugle S, Cowen RK (2013). Ocean acidification alters the otoliths of a pantropical fish species with implications for sensory function. Proc. Natl. Acad. Sci. USA.

[31] Toby BH, Von Dreele RB (2013). GSAS-II: The genesis of a modern open-source all purpose crystallography software package. J. Appl. Crystallogr..

[32] Rietveld HM (1969). A profile refinement method for nuclear and magnetic structures. J. Appl. Crystallogr..

[33] Blanksma C (2009). Effects of water hardness on skeletal development and growth in juvenile fathead minnows. Aquaculture.

[34] Loeppky AR, Anderson WG (2020). Environmental influences on uptake kinetics and partitioning of strontium in age-0 Lake Sturgeon *Acipenser fulvescens*: Effects of temperature and ambient calcium activities. Can. J. Fish. Aquat. Sci..

[35] Takagi Y, Takahashi A (1999). Characterization of otolith soluble-matrix producing cells in the saccular epithelium of rainbow trout (*Oncorhynchus mykiss*) inner ear. Anat. Rec..

[36] Nagasawa H (2013). The molecular mechanism of calcification in aquatic organisms. Biosci. Biotechnol. Biochem..

[37] Ronnestad I, Thorsen A, Finn RN (1999). Fish larval nutrition: A review of recent advances in the roles of amino acids. Aquaculture.

[38] Houlihan D, Hall S, Gray C (1989). Effects of ration on protein turnover in cod. Aquaculture.

[39] Mille T (2016). Diet is correlated with otolith shape in marine fish. Mar. Ecol. Prog. Ser..

[40] Asano M, Mugiya Y (1993). Biochemical and calcium-binding properties of water-soluble proteins isolated from otoliths of the tilapia, *Orecchromis niloticus*. Comp. Biochem. Phys. B.

[41] Coll-Llado C, Giebichenstein J, Webb PB, Bridges CR, de la Serrana DG (2018). Ocean acidification promotes otolith growth and calcite deposition in gilthead sea bream (*Sparus aurata*) larvae. Sci. Rep..

[42] Mosegaard H, Svedäng H, Taberman K (1988). Uncoupling of somatic and otolith growth rates in Arctic char (*Salvelinus alpinus*) as an effect of differences in temperature response. Can. J. Fish. Aquat. Sci..

[43] Lombarte A, Lleonart J (1993). Otolith size changes related with body growth, habitat depth and temperature. Environ. Biol. Fish..

[44] Konopacka-Łyskawa D, Czaplicka N, Łapiński M, Kościelska B, Bray R (2020). Precipitation and transformation of vaterite calcium carbonate in the presence of some organic solvents. Materials.

[45] Kwan GT, Smith TR, Tresguerres M (2020). Immunological characterization of two types of ionocytes in the inner ear epithelium of Pacific Chub Mackerel (*Scomber japonicus*). J. Comp. Physiol. B.

[46] Mosegaard, H. & Titus, R. A. Daily growth rates of otoliths in yolk sac fry of two salmonid species at five different temperatures. In *Proceedings of the Fifth Conference of European Ichthyologists*, 221–227 (1987).

[47] Hüssy K, Mosegaard H, Jessen F (2004). Effect of age and temperature on amino acid composition and the content of different protein types of juvenile Atlantic cod (*Gadus morhua*) otoliths. Can. J. Fish. Aquat. Sci..

[48] Shen SG, Chen F, Schoppik DE, Checkley DM (2016). Otolith size and the vestibulo-ocular reflex of larvae of white seabass *Atractoscion nobilis* at high pCO_2_. Mar. Ecol. Prog. Ser..

[49] Shartau RB, Baker DW, Brauner CJ (2017). White sturgeon (*Acipenser transmontanus*) acid–base regulation differs in response to different types of acidoses. J. Comp. Physiol. B.

[50] Ultsch GR (1996). Gas exchange, hypercarbia and acid–base balance, paleoecology, and the evolutionary transition from water-breathing to air-breathing among vertebrates. Palaeogeogr. Palaeoclimatol. Palaeoecol..

[51] Pracheil BM, Hogan JD, Lyons J, McIntyre PB (2014). Using hard-part microchemistry to advance conservation and management of North American freshwater fishes. Fisheries.

[52] Reimer T (2017). Rapid growth causes abnormal vaterite formation in farmed fish otoliths. J. Exp. Biol..

[53] Flagg, T. A. & Nash, C. F. (eds) A conceptual framework for conservation hatchery strategies for Pacific salmonids. U.S. Department of Commerce. NOAA Technical Memorandum. NMFS-NWFSC. 38, 54 (1999).

[54] Sollner C, Schwarz H, Geisler R, Nicolson T (2004). Mutated Otopetrin 1 affects the genesis of otoliths and the localization of Starmaker in zebrafish. Dev. Genes Evol..

[55] Lundberg YW, Xu Y, Thiessen KD, Kramer KL (2015). Mechanisms of otoconia and otolith development. Dev. Dyn..

[56] Murayama E (2002). Fish otolith contains a unique structural protein, otolin-1. Eur. J. Biochem..

