# Supplementary material for: Influence of ontogenetic development, temperature, and pCO2 on otolith calcium carbonate polymorph composition in sturgeons
Source: Sci Rep. 2021 Jul 6;11:13878. doi: 10.1038/s41598-021-93197-6 (PMC8260795; doi:10.1038/s41598-021-93197-6)
Supplement: Supplementary file 1 — Supplementary Information. [file 41598_2021_93197_MOESM1_ESM.pdf]

Supplementary Information for:

Influence of ontogenetic development, temperature, and *p*CO_2 on otolith calcium carbonate polymorph composition in sturgeons

Alison R. Loeppky[1*], Luke D. Belding[1], Alex R. Quijada-Rodriguez[1], John D. Morgan[2], Brenda M. Pracheil[3], Bryan C. Chakoumakos[4], W. Gary Anderson[1]

[1]Department of Biological Sciences, University of Manitoba, Winnipeg, MB

[2]Department of Resource Management and Protection, Vancouver Island University, Nanaimo, BC

[3]Environmental Sciences Division, Oak Ridge National Laboratory, Oak Ridge, TN

[4]Neutron Scattering Division, Oak Ridge National Laboratory, Oak Ridge, TN

*Corresponding Author: Alison Loeppky

Email: loeppkar@myumanitoba.ca

Supplementary Information Text

SI1 Methods

SI1.1 Fish Rearing. In experiment 1, fertilization and initial seeding techniques of Lake Sturgeon, *Acipenser fulvescens*, were conducted in the Animal Holding Facility at the University of Manitoba in Winnipeg, Canada as described in Loeppky et al. (1). After ~7-10 days of egg incubation, hatched larvae were transferred to multiple 11-litre flow through tanks supplied with dechlorinated City of Winnipeg tap water held at 16ºC with a 12h:12h light to dark photoperiod. Fish were first introduced to freshly hatched brine shrimp nauplii (*Artemia salina*) starting at 10 days post hatch (dph) and fully transitioned to exogenous feeding by 20 dph. At 50 dph fish were then introduced to chironomid larvae (i.e., bloodworms) over the course of several days before being fed solely a bloodworm diet. Throughout larval rearing fish were fed to satiation three times daily at which point all uneaten food and waste was removed from the tanks. Gametes collected from White Sturgeon, *A. transmontanus*, were the artificially fertilized progeny of resident adult males and females at the International Centre for Sturgeon Studies at Vancouver Island University in Nanaimo, Canada. After ~8-11 days of egg incubation, hatched larvae were transferred to 2,000 L tanks supplied with a constant flow of dechlorinated and UV-sterilized Nanaimo municipal freshwater held at 14ºC while photoperiod was determined by an external light sensor. Fish were first introduced to a commercial starter feed (EWOS #0, Cargill Incorporated, Minneapolis, MN, USA) at 5 dph with initial feeding observed at 12 dph and full transition to exogenous feeding occurring at 17 dph. At 49 dph, larger commercial feed was gradually introduced (EWOS #1), although nutritional composition of the feed remained the same. Throughout larval rearing fish were fed to satiation using 24-hr belt feeders with all uneaten food and waste being removed each day.

In experiment 2, Lake Sturgeon egg fertilization was conducted as described in experiment 1. Fertilized eggs were divided into four treatment groups and placed in McDonald tumbling jars for egg incubation. Each treatment was supplied water from separate header tanks corresponding to their pCO_2 levels. The header tanks were injected with a controlled amount of CO_2 regulated by an IKS Aquastar system equipped with pH monitoring electrodes (IKS Aquastar Computer Systeme GmbH, Germany). Temperature in all four treatments was initially maintained at 15ºC in order to ensure the survival of eggs and proper early development of larvae as was determined necessary by a pilot study. At the onset of hatching, larvae were transferred into four 170 L fiberglass aquaria corresponding to their experimental treatment and continued to be supplied water from the header tanks. Given limitations on space and equipment needed to maintain water parameters, fish were required to be reared together in larger aquaria. Great care was taken to ensure all other parameters within and among each treatment tank (e.g. light, stocking density, sampling frequency) in order to minimize tank effects. After 10 dph, temperature in the two 22ºC treatments was gradually increased (0.5ºC/day) until the experimental temperature was achieved. After 150 days, temperature in all four treatment tanks was dropped to 3ºC (0.5ºC/day) to simulate an overwintering period (experimental pCO_2 levels were maintained) that lasted 40 days at which point otolith sampling was conducted. Total alkalinity was measured as described by Sarazin (2) and adjusted for freshwater systems. To calculate pCO_2 in each treatment, measured pH, temperature, and alkalinity were entered into CO_2Calc (3) using K1 and K2 constants for freshwater from Millero (4) with pressure effects on these constants from Millero (5), KSO_4 dissociation constant from Dickson (6) and pH in the NBS scale. Feeding protocols were the same as in Experiment 1, with food being withheld during the overwintering period of the study.

SI1.2 Otolith Sampling. At all sampling points in both Experiment 1 and 2, eight individuals were sacrificed for otolith dissection. Given our studies were novel, we did not have suffient access to previous data to perform an a priori power analysis to determine the ideal/minimum sample size. As such, sample sizes were chosen given limitations to resources required for sample preparation and time required for data quantification on allotted instrument times. However, when post-hoc analyses were conducted with our collected data in G*Power (7), it was determined that the sample sizes in both experiments resulted in powers of 100%. As such, we can concur that that the sample sizes that were chosen provided sufficient statistical power to provide further confidence in our interpretations.

All otolith dissections were conducted at the University of Manitoba following protocols outlined in Loeppky et al. (1). A dissecting microscope (Olympus SZXY7) mounted with polarized lenses (Olympus SZX-PO) was used to aid with the visualization of otoliths during removal. To collect the otoliths, heads were removed at the base of the opercula then sectioned dorsal medially through the sagittal plane from between the occipital cavities to the base of the skull to expose the semicircular canals where the otoliths are housed. Dissected otoliths were then transferred to microscope slides that had a 1 cm^2 5x5 grid covered with a piece of double-sided tape to organize otoliths for transportation to Oak Ridge National Laboratory in Tennessee, USA. Due to the small size and delicate nature of the otoliths, some otoliths were lost during transport.

SI1.3 X-Ray microdiffraction. To quantify the percent polymorph composition by weight of each otolith, X-ray microdiffraction (µXRD) was conducted following protocols outlined in

Loeppky et al. (1). Otoliths were suspended individually in a 300 μm diameter Molecular Dimensions LithoLoop using Paratone oil then placed within a Rigaku XtaLAB PRO diffractometer equipped with graphite monochromated Mo Kα radiation (0.7107 Å) operated at 50 kV and 40 mA, a Dectris PILATUS 200K detector, and Rigaku Oxford Diffraction CrysAlisPro software. The otoliths were then spun around the phi-axis within the X-ray beam to ensure the otolith was fully in the beam and to improve powder diffraction averaging. When placed in an X-ray beam, the X-rays will scatter off the atoms of the otolith producing Debye-Scherrer diffraction patterns (Figure 3b) that are radially integrated to provide bulk information on the structure of the crystals and the identity of the $CaCO_3$ polymorphs.

SI1.4 Blood pH measurements. Because of the small size of fish, blood samples in Experiment 2 were collected by severing the tail of euthanized fish to expose the caudal sinus and drawing blood using a heparinized capillary tube. The blood was then ejected from the capillary tube using a syringe and transferred into 1.5 mL eppendorph vials. Blood samples were floated in a 3°C water bath (Julabo F25-MC, Allenton, PA, USA) and pH was measured with a thermostated micro pH electrode (InLab Micro, Mettler Toledo, Ohio, USA). To correct for gas exchange that may have occurred during the expelling of the blood from the capillary tube, larger Lake Sturgeon (3 years) were acclimated to the same experimental pCO_2 levels for three weeks at 3ºC in duplicate 170 L tanks (n = 6 fish/treatment). Fish were lightly anesthetized in an anesthetic bath that was created using 150 $mg.L^{-1}$ tricane methanosulphate (MS-222) buffered with equal volumes of sodium bicarbonate in their respective treatment waters. Blood samples were drawn from the caudal sinus using a syringe then transferred to an eppendorf tube so that blood pH could be measured immediately. A capillary tube was then used to draw a small sample of blood

then expelled using a syringe to mimic the measuring of the age-0 fish. The percent change in blood pH from the syringe method and capillary method was consistent across treatments and thus was used to correct the original blood pH data collected from the experimental fish using the capillary method.

SI1.5 Otolith imaging. To compare otolith size in Experiment 2, otoliths were imaged using a dissecting microscope (Olympus SZXY7) at 10x magnification. Otolith area was calculated using Fiji for ImageJ software (http://rsb.info.nih.gov/i) by converting images to 8-bit then adjusting the threshold to isolate the outline of the otolith. The function Analyze Particles was then used to calculate the area of the otolith.

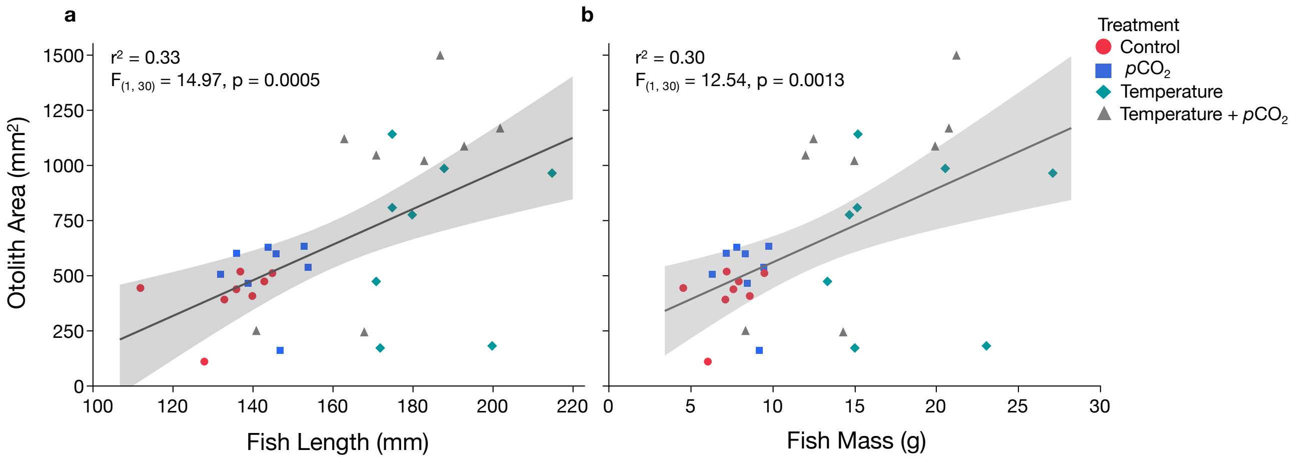

Fig. S1. Regression analyses examining the relationship of otolith area (mm^2) and (**a**) fish length (mm) and (**b**) fish mass (g) with 95% confidence intervals shaded in grey. Rearing treatment is indicated by red circles (control, 15ºC, 1000 µatm pCO2), blue squares (pCO2, 15ºC, 2500 µatm pCO2), teal diamonds (temperature, 22ºC, 1000 µatm pCO2), and grey triangles (temperature + pCO2, 22ºC, 2500 µatm pCO2). The coefficient of determination and significance are presented for each regression.

Table S1. Mean (±s.e.m.) environmental rearing conditions within each rearing location in Experiment 1 (ontogenetic effects) and rearing period in Experiment 2 (environmental effects).

Experiment 1 – Ontogenetic effects

Rearing Location	Species	Temperature (°C)	pH	Alkalinity ($mg.L^{-1}$)	Hardness ($mg.L^{-1}$)
Winnipeg, MB	Lake Sturgeon	16.3 (±0.08)	7.75*	68.3*	80.7*
Nanaimo, BC	White Sturgeon	13.8 (±0.01)	7.58*	32.1*	32.0*

Experiment 2 – Environmental effects

Rearing Period	Treatment	Temperature (°C)	pH	Alkalinity ($mg.L^{-1}$)	pCO_2 (µatm)
Summer/fall rearing period	Control	15.6 (±0.03)	7.78 (±0.07)	121.9 (±1.9)	1096.9 (±136.55)
	pCO_2	15.7 (±0.06)	7.44 (±0.06)	122.5 (±1.3)	2644.2 (±109.54)
	Temperature	22.4 (±0.58)	8.00 (±0.03)	123.1 (±1.2)	1019.7 (±41.90)
	Temperature + pCO_2	22.5 (±0.58)	7.44 (±0.08)	121.4 (±1.0)	2365.3 (±116.64)
Overwintering period	Control	2.9 (±0.10)	7.64 (±0.10)	120.5 (±1.8)	1041.2 (±46.30)
	pCO_2	2.9 (±0.17)	7.04 (±0.06)	119.8 (±1.5)	2594.0 (±78.10)
	Temperature	2.8 (±0.09)	7.48 (±0.06)	121.4 (±7.9)	1088.6 (±81.68)
	Temperature + pCO_2	2.9 (±0.12)	7.07 (±0.03)	120.4 (±2.4)	2648.4 (±117.82)

* Indicates measurements were obtained from City of Winnipeg and Nanaimo water quality reports (winnipeg.ca; nanaimo.ca)

Table S2. Statistical results of general linear models from both the ontogenetic and environmental effects experiments. In Experiment 1, otolith (i.e, sagitta, lapillus) polymorph composition were the response variables with species (i.e., Lake Sturgeon, White Sturgeon) and developmental stage (i.e., days post hatch, dph) as fixed independent variables. Days 12 and 14 dph were not included in the model for lapilli otoliths because they were not sampled from Lake Sturgeon. In Experiment 2, response variables were % polymorph composition (i.e., vaterite, aragonite, calcite), normalized (z-score) otolith area (mm^2), fish length (mm), fish mass (g), and blood pH with experimental temperature (°C) and pCO_2 (μatm) as fixed independent variables. P-values <0.05 indicate significant differences and are bolded.

Experiment 1 – Ontogenetic effects

Response Variable	Independent Variable	df	df_{error}	F ratio	p-value
Sagitta	Species	1	129	33.59	**<.0001**
	dph	10	129	15.71	**<.0001**
	Species x dph	10	129	3.89	**0.0001**
Lapillus	Species	1	105	0.55	0.45
	dph	8	105	14.83	**<.0001**
	Species x dph	8	105	8.33	**<.0001**

Experiment 2 – Environmental effects

Response Variable	Independent Variable	df	df_{error}	F ratio	p-value
Vaterite	Temperature	1	28	1.40	0.25
	pCO_2	1	28	0.24	0.63
	Temperature x pCO_2	1	28	1.67	0.21
Aragonite	Temperature	1	28	0.08	0.25
	pCO_2	1	28	0.003	0.95
	Temperature x pCO_2	1	28	0.41	0.53
Calcite	Temperature	1	28	10.07	**0.004**
	pCO_2	1	28	1.88	0.18
	Temperature x pCO_2	1	28	1.00	0.17
Otolith area	Temperature	1	28	11.18	**0.002**
	pCO_2	1	28	2.51	0.12
	Temperature x pCO_2	1	28	0.54	0.47
Fish length	Temperature	1	28	67.49	**<.0001**
	pCO_2	1	28	0.02	0.89
	Temperature x pCO_2	1	28	3.31	0.08
Fish mass	Temperature	1	28	48.75	**<.0001**
	pCO_2	1	28	0.64	0.43
	Temperature x pCO_2	1	28	2.09	0.16
Blood pH	Temperature	1	28	4.05	0.053
	pCO_2	1	28	31.00	**<.0001**
	Temperature x pCO_2	1	28	0.21	0.65

Table S3. Mean (± s.e.m.; n = 8/treatment) blood pH measurements of age-0 Lake Sturgeon, *Acipenser fulvescens*, at sampling (260 days post hatch) within each rearing treatment in Experiment 2 (i.e., control [15ºC, 1000 µatm pCO_2], pCO_2 [15ºC, 2500 µatm pCO_2], temperature [22ºC, 1000 µatm pCO_2], temperature + pCO_2 [22ºC, 2500 µatm pCO_2]).

Rearing Treatment	Blood pH
Control	7.78 (±0.03) [a]
pCO_2	7.96 (±0.03) [b]
Temperature	7.84 (±0.02) [a]
Temperature + pCO_2	7.98 (±0.03) [b]

[a,b] indicate results for post-hoc Tukey HSD tests among treatments. Values not sharing the same letter are significantly different ($p<0.05$).

SI References

Sample References:

1. A. R. Loeppky, B. C. Chakoumakos, B. M. Pracheil, W. G. Anderson, Otoliths of sub-adult Lake Sturgeon *Acipenser fulvescens* contain aragonite and vaterite calcium carbonate polymorphs. *J. Fish Biol.* 94, 810-814 (2019).
2. G. Sarazin, G. Michard, F. Prevot, A rapid and accurate spectroscopic method for alkalinity measurements in sea water samples. *Water Res.* 33, 290-294 (1999).
3. L. Robbins, M. Hansen, J. Kleypas, S. Meylan, CO2calc—a user-friendly seawater carbon calculator for Windows, Max OS X, and iOS (iPhone). *US geological survey open-file report* 1280, 2010 (2010).
4. F. J. Millero, The thermodynamics of the carbonate system in seawater. *Geochim. Cosmochim. Acta* 43, 1651-1661 (1979).
5. F. J. Millero, "Influence of pressure on chemical processes in the sea" in Chemical oceanography. (Elsevier, 1983), pp. 1-88.
6. A. G. Dickson, Standard potential of the reaction: AgCl (s)+ 12H2 (g)= Ag (s)+ HCl (aq), and and the standard acidity constant of the ion HSO4− in synthetic sea water from 273.15 to 318.15 K. *J. Chem. Thermodyn.* 22, 113-127 (1990).
7. F. Faul, E. Erdfelder, A. -G. Lang, & A. Buchner, G*Power 3: A flexible statistical power analysis program for the social, behavioral, and biomedical sciences. *Behav. Res, Methods*, 39, 175-191 (2007).
